# Kraft Lignin Decomposition by Forest Soil Bacterium *Pseudomonas kribbensis* CHA-19

**DOI:** 10.4014/jmb.2406.06021

**Published:** 2024-07-19

**Authors:** Dockyu Kim, Han-Woo Kim, Hyoungseok Lee

**Affiliations:** 1 Division of Life Sciences, Korea Polar Research Institute, Incheon 21990, Republic of Korea; 2 Department of Polar Sciences, University of Science and Technology, Incheon 21990, Republic of Korea

**Keywords:** Bacterial peroxidase, bacterial laccase, biocatalyst, biodegradation, kraft lignin, *Pseudomonas*

## Abstract

Identification of the biochemical metabolic pathway for lignin decomposition and the responsible degradative enzymes is needed for the effective biotechnological valorization of lignin to renewable chemical products. In this study, we investigated the decomposition of kraft lignin by the soil bacterium *Pseudomonas kribbensis* CHA-19, a strain that can utilize kraft lignin and its main degradation metabolite, vanillic acid, as growth substrates. Gel permeation chromatography revealed that CHA-19 decomposed polymeric lignin and degraded dehydrodivanillin (a representative lignin model compound); however, the degradative enzyme(s) and mechanism were not identified. Quantitative polymerase chain reaction with mRNAs from CHA-19 cells induced in the presence of lignin showed that the putative genes coding for two laccase-like multicopper oxidases (LMCOs) and three dye-decolorizing peroxidases (DyPs) were upregulated by 2.0- to 7.9-fold compared with glucose-induced cells, which indicates possible cooperation with multiple enzymes for lignin decomposition. Computational homology analysis of the protein sequences of LMCOs and DyPs also predicted their roles in lignin decomposition. Based on the above data, CHA-19 appears to initiate oxidative lignin decomposition using multifunctional LMCOs and DyPs, producing smaller metabolites such as vanillic acid, which is further degraded via *ortho*- and *meta*-ring cleavage pathways. This study not only helps to better understand the role of bacteria in lignin decomposition and thus in terrestrial ecosystems, but also expands the biocatalytic toolbox with new bacterial cells and their degradative enzymes for lignin valorization.

## Introduction

Lignocellulose is composed of cellulose, hemicellulose, and lignin, and is the largest organic polymer reservoir in the ecosystem. As a major component (15%–30%) of lignocellulosic biomass, lignin is a complex and heterogeneous aromatic polymer found in the cell walls of plants and has the potential to be a valuable resource for producing renewable chemicals [1]. Ongoing research into the extraction and modification of lignin for various applications contributes to the development of more sustainable and environmentally friendly technologies. Lignin is a byproduct (approximately 50 million tons annually) of the pulp and paper industry, which in combination with commercial cellulosic ethanol production helps provide an oversupply of lignin [2]. Therefore, lignin has gained attention owing to its potential industrial applications in producing biofuels, adhesives, and other value-added chemicals.

Lignin valorization refers to the extraction of compounds such as vanillin, flavor, and other value-added chemicals [3] from lignin, and is thus considered a key process in the successful development of lignocellulosic biorefineries. Several strategies have emerged for lignin valorization, including chemical, biological, and thermochemical processes, which are necessary for taking full advantage of lignin’s potential as a natural resource. However, owing to its polymeric structural complexity and high molecular weight, lignin is insoluble in water and resistant to microbial and enzymatic degradation, making lignin breakdown a challenging task. Biological conversion processes using microbial cells (bacteria or fungi) or their extracellular enzymes can break down lignin into simpler molecules and valuable products such as biofuels or bioplastics [4].

Soil fungal basidiomycetes, especially white-rot fungi, have been well characterized to oxidatively decompose lignin using extracellular lignin peroxidases, laccases, and manganese peroxidases, which can break down the complex structure of lignin. The resulting small aromatic and aliphatic compounds are completely degraded by the surrounding bacteria. For example, vanillic acid, the main aromatic metabolite resulting from enzymatic lignin cleavage [5], is converted to protocatechuate, which is further degraded via *ortho* and/or *meta* cleavage pathways. Many lignin-degrading bacteria in several genera (*Rhodococcus*, *Amycolatopsis*, *Pseudomonas*, *Ochrobactrum*, *Bacillus*, and *Streptomyces*) were isolated from various natural and industrial environments [6]. From them, peroxidases [7], dye-decolorizing peroxidases (DyPs) [8-10], laccases [11], laccase-like enzymes [1, 12, 13], multicopper oxidases (MCOs) [14], and β-etherases [15, 16] have been detected and assumed to cleave C–C and C–O–C linkages within lignin. Although fungal ligninolytic capability is much higher than that of bacteria, some bacterial strains can be explored for lignin valorization because of their rapid growth, easy genetic manipulation, and metabolic versatility with diverse catalytic enzyme systems and pathways. Particularly, bacterial laccases are more thermostable and resistant to harsh conditions (alkali pH and salinity), and their overproduction of recombinant enzymes is accomplished relatively easier than with their fungal counterparts [1]. Therefore, the bacterial strains with higher ligninolytic capability and efficient bioconversion systems are prerequisite for successful lignin valorization [6].

Here, we aimed to characterize a potential lignin-degrading bacterium isolated from forest organic soil that exhibited rapid growth in a medium containing kraft lignin as the sole carbon source. The lignin-degrading capability of the isolate was determined physiologically and spectrophotometrically, and its putative ligninolytic enzymes (DyPs and LMCOs) responsible for the initial lignin decomposition were analyzed using mRNA-targeted quantitative polymerase chain reaction (PCR) and computer-aided functional prediction methods. This bacterial isolate is valuable for lignin valorization because of its growth capability, decomposition activity, and cooperative reaction between multiple ligninolytic enzymes.

## Materials and Methods

### Growth Test on Polymeric Lignin and Lignin Metabolite

*Pseudomonas kribbensis* CHA-19 (KCTC 72262) was previously isolated from forest organic soil in New Jersey, USA, owing to its ability to degrade polymeric humic acids (HA). The draft genome sequence of strain CHA-19 was analyzed to identify the genes involved in HA decomposition, and its HA-degradative pathway was proposed previously [17]. CHA-19 was inoculated into mineral salts basal (MSB) broth containing 20 mM glucose (MSB+Glu) and incubated at 28°C for 2 days with shaking. After culturing, the glucose adhering to the cell surface and remaining in the culture medium was removed by centrifuging (8,000 ×*g* for 20 min) and washing the cell pellet with new MSB broth. The cell suspension in MSB was transferred to 50 ml of fresh MSB in an Erlenmeyer flask (250 ml) at an absorbance at 600 nm of 0.1. Kraft lignin (Sigma-Aldrich, cat. no. 370959, USA) and vanillic acid (Sigma-Aldrich, cat. no. H36001) were completely dissolved in 1.0 N NaOH (10% stock solution) and distilled water with a small amount of NaOH (250 mM stock solution), respectively, and added to a flask at final concentrations of 0.05% (lignin) and 3 mM (vanillic acid). During culturing at 28°C for 25 days, at indicated time intervals, 100 μl of the culture was diluted in MSB, and 100 μl of this dilution was plated on MSB+Glu. After incubation at 28°C, the number of colony-forming units (CFU) per milliliter was calculated.

### Decomposition of Polymeric Lignin

Strain CHA-19 was grown in 80 ml MSB+Glu at 28°C for 3 days with shaking, and the cells were harvested by centrifugation (8,000 ×*g* for 20 min). The pellet was resuspended in 40 ml MSB and transferred to an Erlenmeyer flask (250 ml). Kraft lignin was then added to the flask at a final concentration of 0.05%. The control was prepared without the inoculation of the cell pellet. The flasks were incubated with shaking at 28°C, and the structural changes in lignin owing to the decomposition activity of CHA-19 were analyzed by gel permeation chromatography (GPC), as described below.

After 15 days of incubation, a small portion (1.0 ml) of the culture was centrifuged (10,000 ×*g* for 10 min). The supernatant was filtered through a hydrophilic membrane (0.2 μm), and the filtrate (20.0 μl, 0.5 mg of lignin/ml) was separated using Shodex OHpak SB-G 6B guard (6.0 mm ID × 50 mm length), SB-804 HQ (8.0 × 300 mm), and SB-805 HQ (8.0 × 300 mm) columns tandemly connected to an Agilent Technology 1200 HPLC (high-performance liquid chromatography) System. The flow rate of the mobile phase (degassed water) was 0.5 ml/min, and the GPC eluates were examined with a refractive index detector. A set of Shodex standard pullulan samples (kit P-82; 9,600–642,000 Da) was used as the molecular weight reference.

### Degradation of Lignin Model Compound

CHA-19 cells were grown in 200 ml of MSB+Glu at 28°C. After 3 days of culturing, the cells were harvested, washed, resuspended in 20 ml MSB, and transferred to an Erlenmeyer flask following the same procedures as in the polymeric lignin degradation experiment. Two representative lignin model compounds, dehydrodivanillin (DDV; BLDpharm, cat. no. BD323471; 250 mM stock solution in ethanol) and guaiacylglycerol β-guaiacyl ether (GGE; A2B Chem, cat. no. AB74526; 250 mM stock solution in dimethyl sulfoxide), were added to the flask at a final concentration of 2.5 mM. The control was prepared without the inoculation of the cell pellet. After shaking incubation at 28°C for 9 days, a small portion (1.0 ml) of the culture was centrifuged and filtered through a hydrophilic membrane. The filtrate (5.0 μl) was separated using a Phenomenex Kinetex 5 μm C18 LC column (4.6 × 250 mm) connected to an Agilent Technology 1200 HPLC System. The set parameters were as follows: flow rate, 0.5 ml/min; wavelength, 280 nm; gradient of mobile phase (acetonitrile with 0.1% formic acid/water with 0.1% formic acid), initial 30:70%, 90:10% for 15 min, 100:0% for 10 min, 100:0% for 5 min, for a total time of 30 min/run.

### Cell Induction and Total RNA Extraction

CHA-19 preculture (50 ml) in MSB+Glu was transferred to 450 ml MSB+Glu and cultured at 28°C for 3 days. The grown cells were centrifuged (8,000 ×*g* for 20 min), washed with fresh MSB, and resuspended in 200 ml of MSB. The cell suspension was split into 50-ml aliquots, and each was induced by glucose (3 mM), vanillic acid (3 mM), or lignin (0.05%) by incubating with shaking at 28°C for 5 or 24 h in the dark. The induced cells were pelleted (8,000 ×*g*, 20 min, 4°C) and stored at -80°C until required. Total RNA from each sample was extracted using the easy-BLUE reagent (iNtRON Biotechnology, Republic of Korea) according to the manufacturer's instructions. The resulting RNA pellet was dissolved in RNase-free water and purified on an Amersham MicroSpin S-400 HR column (Cytiva, USA) to remove any remaining PCR inhibitor (lignin).

### Target Gene mRNA Quantification Using Quantitative PCR

After DNA was removed from the total RNA extracts using DNase I (Thermo Fisher Scientific, USA), reverse transcription was performed using a qPCRBIO cDNA Synthesis Kit (PCR Biosystems, UK). The resulting cDNAs for putative LMCO, DyP, and aromatic ring-cleavage dioxygenases were amplified using the following primer pairs: (5'→3') TFH80052 (221 bp; Forward, GCCGTTACAGGAACAGGAAA; Reverse, TCTTGTTCA CACCGTTGGAG), TFH80975 (242 bp; GCCGAAGACAAGGACAACAT; CCGTCTTCCAGACCACTCAT), TFH81056 (242 bp; GCGAAGAGGTGAAAATCCTG; TTATCGCTCTGCCAGCTCTT), TFH77958 (197 bp; CCTTGAGCCTGAAGAACTGG; GCGATAGGTCAGGGTGTTGT), TFH78995 (179 bp; CAGCGAAAAGGA CAATCCAT; TGAGGTGCAGTTCGATGGTA), TFH82233 (175 bp; GACCCGAATTTCGAAGGTTT; AAT AAATCCGCGTCAGCAAG), TFH78177 (238 bp; ATTTTCGCAATCCCAAACTG; TAAGTGTCATCGTCG GTTCG), and 16S rRNA (204 bp; AAGCAACGCGAAGAACCTTA; CACCGGCAGTCTCCTTAGAG). Real-time quantitative PCR was performed on an ABI 7500 Real-Time PCR System (Thermo Fisher Scientific) using 2× qPCRBIO SyGreen Mix Lo-ROX (PCR Biosystems). Thermal cycling conditions were as follows: 95°C for 3 min, followed by 40 cycles of 95°C for 10 s, and 60°C for 30 s. The expression level of each target gene was normalized to the 16S rRNA gene as an endogenous control, and the fold-change value was calculated between vanillic acid or lignin-induced cells and glucose-induced cells using the 2-ΔΔCt method.

## Results and Discussion

### Growth Tests on Polymeric Lignin and Its Metabolite

**Fig. 1:**
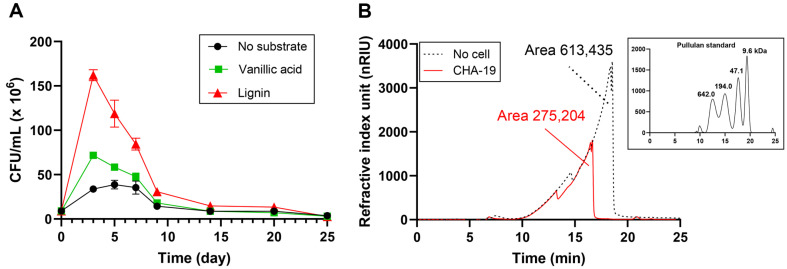
(A) Viability over incubation time of *Pseudomonas kribbensis* CHA-19 at 28°C for 25 days. (B) Gel permeation chromatography elution profile of polymeric lignin after incubation with CHA-19 resting cells at 28°C for 15 days.

In this study, no additional carbon source was available in the MSB medium; thus, strain CHA-19 could only utilize the substrate (lignin or vanillic acid) provided as the sole carbon source. To determine the growth of CHA-19 cells, CFU values were determined over a 25-day culture period. After cultivation for 3 days, CHA-19 cells reached the highest growth level (1.6 × 10^8^ CFU/ml) on polymeric lignin and a lower level (0.7 × 10^8^ CFU/ml) on vanillic acid (a lignin-degradative metabolite), whereas they reached to 0.3 × 10^8^ CFU/ml in the absence of growth substrate (Fig. 1A). Compared with previous studies on bacterial growth (10^5^–10^9^ CFU/ml) in lignin-supplemented medium [6], CHA-19 was confirmed to be a genuine lignin degrader.

Following the confirmation of the capacity of CHA-19 to use lignin as a growth substrate, lignin decomposition was examined using chromatography. After 15 days of incubation of CHA-19 cells with lignin, a small portion of the culture was centrifuged, and the supernatant was analyzed by GPC. Intact lignin, which was not treated with CHA-19 cells (control), was separated at a retention time (RT) of 5–20 min and determined to be 9.6–642.0 kDa in size, compared with the GPC elution profile of the pullulan standard. For the CHA-19-inoculated test sample, the lignin content significantly decreased by 45% (integrated peak area of 275,204 at 7–19 min) compared with that of the control (peak area of 613,435 at 5–20 min), which is indicative of the loss of the lignin fraction owing to CHA-19 decomposition (Fig. 1B).

### Lignin Model Compound Degradation

**Fig. 2:**
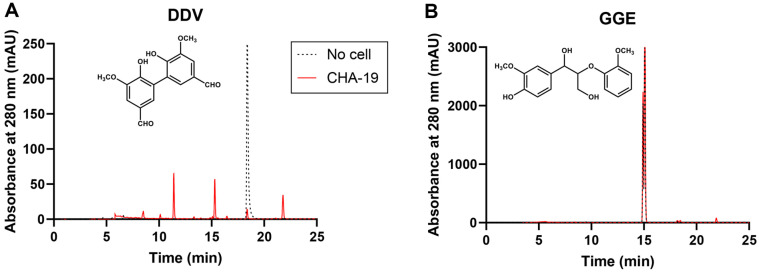
High-performance liquid chromatography elution profile of metabolites formed after (**A**) dehydrodivanillin (**DDV**) and (**B**) guaiacylglycerol β-guaiacyl ether (**GGE**) incubation with *Pseudomonas kribbensis* CHA-19 resting cells at 28°C for 9 days.

To characterize the lignin decomposition strategies, the cleavage of certain lignin linkages was examined using the lignin model compounds, DDV and GGE. After DDV bioconversion using CHA-19 resting cells for 9 days, the reaction solution was analyzed using HPLC. As shown in Fig. 2A, the DDV peak at an RT of 18.3 min almost disappeared, producing novel distinct peaks at RTs of 11.4, 15.3, and 21.8 min. This suggests that the presence of an enzyme system involved in DDV degradation and that the novel peaks are the products of the enzymatic degradation reaction. The 11.4-min peak area largely increased with incubation time, whereas the others rarely changed in their RT and peak area (data not shown). These more hydrophilic novel compounds at 11.4 and 15.3 min were assumed to be vanillic acid (9.7 min) and vanillin (12.9 min), possible metabolites through bacterial DDV bioconversion [18]; however, their RTs were not consistent with that of respective authentic compound. The DDV-derived metabolites, vanillic acid and vanillin, might have been converted to unknown dead-end products, which were not further catabolized by CHA-19. In contrast to DDV, GGE incubated with CHA-19 cells rarely changed their RT and peak area after 9 days of reaction, compared with the no-cell control (Fig. 2B), which indicated that the enzymatic system for GGE degradation was either absent or expressed at a very low level in CHA-19 cells.

The β-aryl ether (including GGE) and biphenyl (including DDV) structures are dimeric compounds that are dominantly found in native polymeric lignin, accounting for 51% and 11%, respectively [19]. Each has been used as a model for characterization of different enzymatic reactions involving lignin decomposition. The distinct enzymes and pathways involved in the degradation of β-aryl ether (LigDD2, LigEF, and LigG) and biphenyl (LigX, LigZ, LigY, and LigWW2) structures to vanillin and/or vanillic acid are well identified and characterized in *Sphingomonas paucimobilis* SYK-6 [19]. The resulting vanillin and vanillic acid can be converted to each other by a reversible enzymatic reaction [18]. Considering that CHA-19 is able to grow on vanillic acid, low expressions of the catabolic enzymes for GGE degradation pathway seem to be more reasonable for CHA-19.

### Identification of Lignin-Degradative Enzyme Genes Using Bioinformatic Analysis

Many lignin-degrading bacteria, including diverse genera, utilize oxidative enzymes such as DyPs and LMCOs to cleave C–C and C–O–C linkages within polymeric lignin or lignin model compounds. Genes for either DyPs [8] or LMCOs [20] frequently exist in multiple forms; however, the precise roles of different isoenzymes or genes remain largely unknown. Some bacteria were identified to possess multiple genes for both the enzymes, possibly for efficient lignin oxidation in their single host [1, 12, 14].

**Fig. 3:**
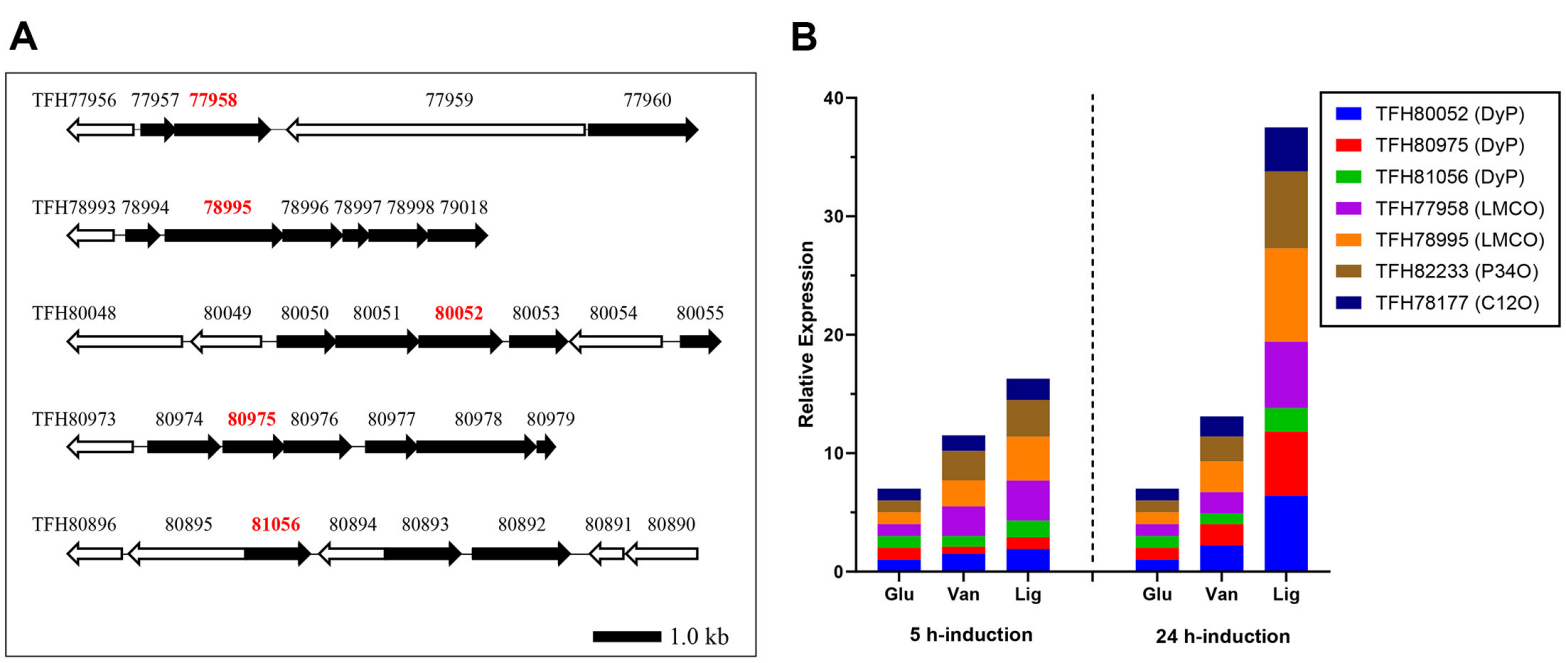
(A) Operonic structure of putative genes for initial lignin oxidative enzymes and neighboring proteins on *Pseudomonas kribbensis* CHA-19 genome. The gene IDs for dye-decolorizing peroxidase (DyP; TFH80052, TFH80975, and TFH81056) and laccase-like multicopper oxidases (LMCO; TFH77958 and TFH78995) are shown in red letters. (**B**) Comparison of mRNA expression levels of target genes in CHA-19 cells induced by vanillic acid (Van) or lignin (Lig) with those by glucose (Glu). mRNAs were extracted from the CHA-19 cells after 5- or 24-h induction at 28°C.

Gene annotation of the CHA-19 genome (approximately 6.4 Mb) revealed 5,737 coding sequences, of which several putative HA-degradative genes were detected and used to propose an HA-degradation pathway [17]: LMCOs (GenBank Accession Nos. TFH77958 and TFH78995), DyPs (GenBank Accession Nos. TFH80052, TFH80975, and TFH81056), protocatechuate 3,4-dioxygenase (P34O; GenBank accession no. TFH82233), and catechol 1,2-dioxygenase (C12O; GenBank Accession No. TFH78177). Based on the structural similarity between HA and lignin and the presence of main lignin components (especially guaiacol) in the HA structure, the LMCO-and DyP-annotated genes were assumed to be involved in the initial lignin oxidative degradation, with P34O- and C12O-annotated genes performing aromatic ring cleavage of lignin-derived small metabolites. The operonic structures of these genes and other neighboring genes and their predicted functions are depicted and summarized in Fig. 3A and Table 1. Several studies reported on bacterial laccase, LMCOs, and DyPs that initiate the lignin decomposition via β-O-4 ether bond cleavage in lignin [10, 21, 22]. Bacterial DyPs were characterized to cleave C_α_–C_β_ bond in β-aryl ether structures (*e.g.*, GGE) within lignin polymer, finally producing small-molecular-weight, lignin-derived compounds, such as guaiacol and vanillin as main metabolites [8, 23]. Some bacterial lignin catabolic pathways lead to the production of vanillin or its oxidation product vanillic acid, which is converted to protocatechuic acid and can be degraded via oxidative *ortho*-ring cleavage using P34O. In an alternative pathway, vanillic acid is decarboxylated to guaiacol, which is converted to catechol, the aromatic ring of which can be cleaved via *meta*-ring reacting with C12O.

### Targeted mRNA Analysis for Lignin Decomposition

To quantitatively analyze the expression levels of specific genes predicted to be involved in lignin decomposition and lignin metabolite degradation, the putative LMCO, DyP, P34O, and C12O genes were induced in CHA-19 cells by incubation with lignin or vanillic acid. Subsequently, real-time quantitative PCR was performed using the mRNA from individually induced cells. As shown in Fig. 3B and Table 2, when CHA-19 cells were induced with lignin for 5 h, the mRNA expression levels of two LMCO genes were significant (expression fold change, 3.4–3.7) compared with glucose-induced cells, and the fold change increased considerably to 5.6–7.9 after 24-h induction. The expression patterns of the P34O and C12O genes were similar to those of the LMCO genes (*i.e.*, higher expression with an increase in induction time). Three DyP genes were more significantly expressed (fold change, 2.0–6.4) after 24 h than after 5 h. In summary, all genes tested were distinctly expressed, indicating their involvement in polymeric lignin decomposition. Remarkably, the mRNA expression levels of the LMCO and P34O genes in vanillic acid-induced cells remained high (expression fold change, >1.8) from 5 to 24 h, which is indicative of their main functions in lignin decomposition.

### Functional Prediction of DyPs and LMCOs for Initial Lignin Decomposition

**Fig. 4:**
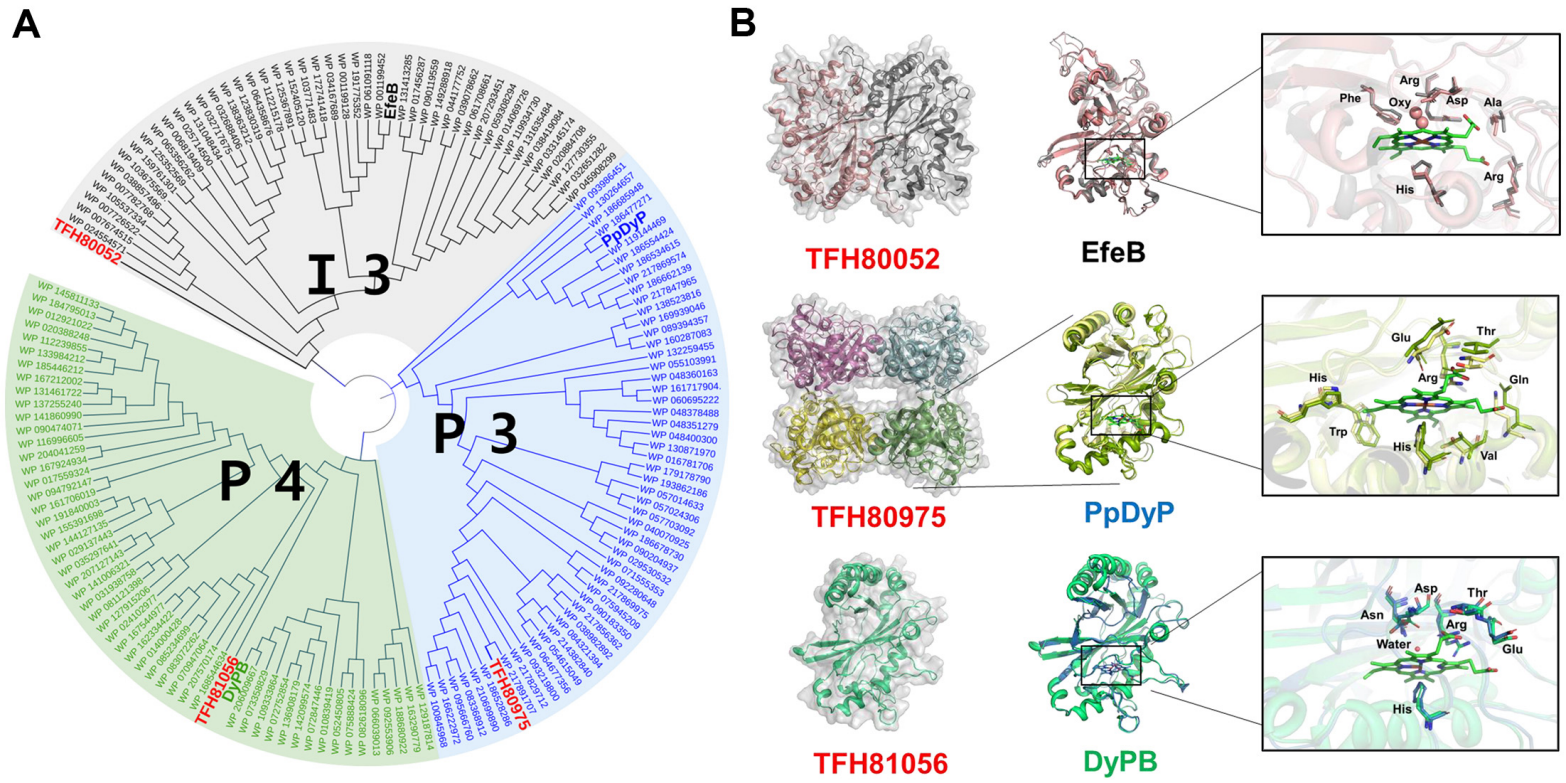
Phylogenetic tree and structure models of three DyPs expressed in *Pseudomonas kribbensis* CHA-19. (**A**) Phylogenetic analysis of the CHA-19 DyPs (TFH80052, TFH80975, and TFH81056). Multiple sequence alignment of the proteins was performed using ClustalW. The sequences represented by NCBI reference number were used for phylogenetic analysis. The phylogenetic tree construction from the aligned sequences was performed using MEGA 11. The phylogenetic tree was visualized using iTOL version 6. EfeB from *Escherichia coli*, PpDyP from *Pseudomonas putida*, and DyPB from *Rhodococcus jostii* RHA1 are shown as representative enzymes of the subclasses I3, P3, and P4, respectively. (**B**) Overall structure of the CHA-19 DyPs predicted by ColabFold platform. Left, predicted multimer structure of each protein; middle, superimposition of the predicted DyPs structures with their corresponding proteins, EfeB (PDB ID: 3O72), PpDyP (PDB ID: 7QYQ), and DyPB (PDB ID: 3QNS); right, zoom-in view of the heme active site. The heme site structures are shown as stick and sphere models. The water molecule is depicted as a cyan sphere. The active site residues have hydrogen bond interaction with heme molecule in PDB data of DyPs.

To gain a deeper insight into the function of LMCOs and DyPs from CHA-19, we used local alignments using well-known databases and computer-aided enzyme modeling. DyP enzymes belong to a family of heme peroxidases, which are mainly derived from fungal sources and have wide substrate specificity. DyPs have been found in a variety of organisms, functioning in a wide cellular distribution range, from intracellular to extracellular, including the periplasmic space. In the RedoxiBase database [24], DyPs are categorized as classes A, B, C, and D by primary sequence homology. Recently, these DyP groups were reclassified as classes I, P, and V, respectively, using the multiple three-dimensional alignment tool [25]. In the new classification, the three DyPs (TFH80052, TFH80975, and TFH81056) from CHA-19 were classified into subclasses I3, P3, and P4, respectively (Fig. 4). Class I DyPs found in bacteria have twin arginine translocation (Tat) signal sequences that are exported into the periplasmic space. TFH80052 also has a predicted N-terminal Tat sequence, which has a high sequence identity with EfeB from *Escherichia coli*. The Efe system is a mechanism for siderophore-independent iron uptake induced by low pH or iron concentrations [26]. On the CHA-19 genome, we also found an EfeO homologue (TFH80051) related to the Efe system (Table 1). EfeB has bifunctional catalytic properties, with guaiacol peroxidase activity at low pH [27]. Lignin decomposition by class I DyPs has been reported for bacterial enzymes, such as DyPA from *Pseudomonas fluorescens* pf-5 [9], TfuDyP from *Thermobifida fusca* [28], and SviDyP from *Saccharomonospora viridis* DSM43017 [29]. Although TFH80052 and TFH81056 have putative signal peptides, we could not identify a signal peptide in TFH80975 using SignalP analysis [30]. TFH80975 is highly similar to PpDyP (DyP from *Pseudomonas putida*), which has high catalytic activity against lignin-related phenolic compounds. Recombinant PpDyP was expressed as a homotetramer. TFH81056 contains the Sec signal sequence and has 59% identity with DyPB from *Rhodococcus jostii* RHA1, which catalyzes the oxidative degradation of kraft lignin [8]. RHA1 contains two DyPs (DyPA and DyPB). DyPA is highly similar to TFH80052, which has been identified as class I. DyPB is expressed as a cargo enzyme in encapsulin, a self-assembling protein nanocompartment [31]. However, we found no encapsulation-related genes in the vicinity of TFH81056 in the CHA-19 genome.

**Fig. 5:**
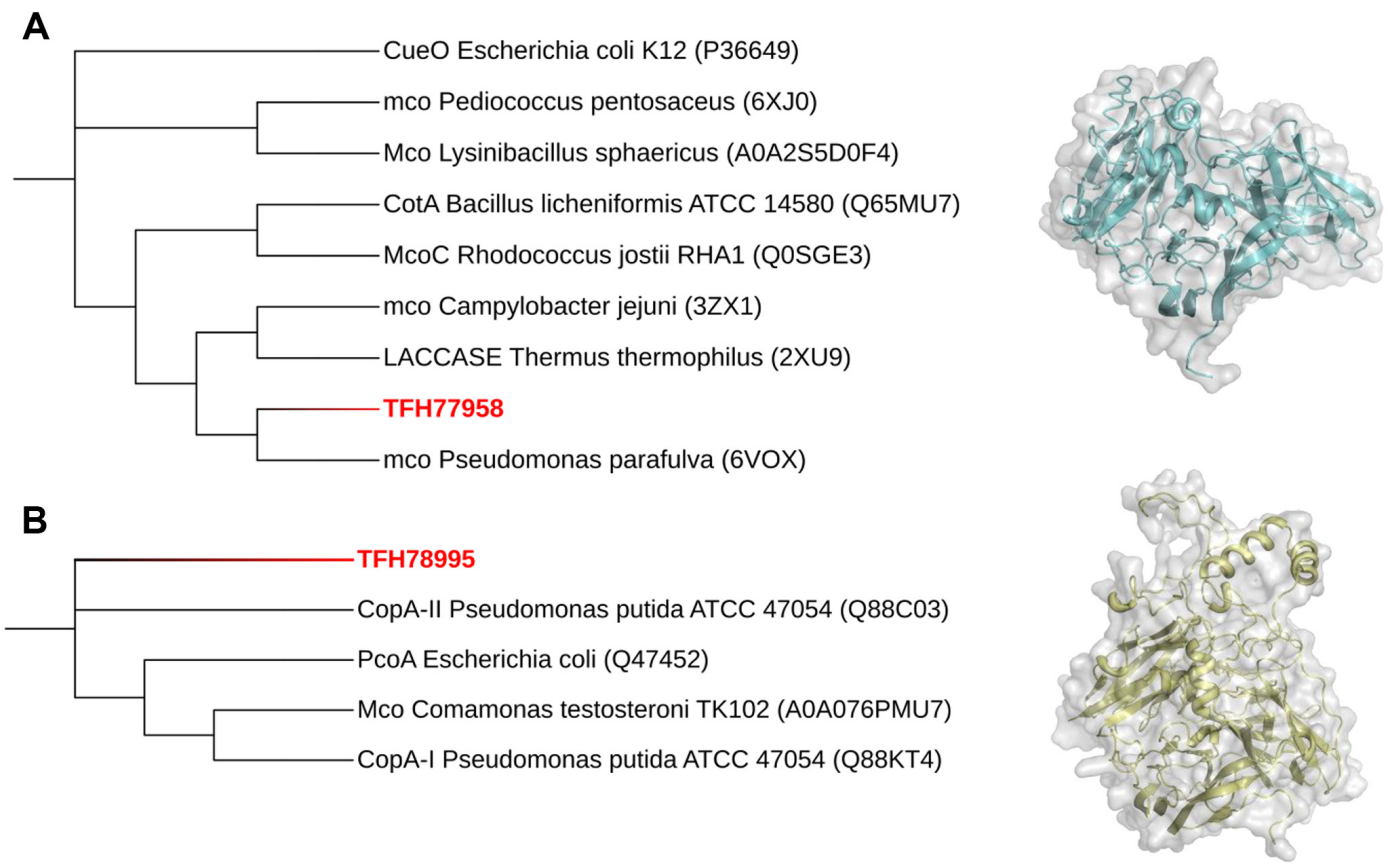
Phylogenetic analysis and structure prediction of two LMCOs expressed in *Pseudomonas kribbensis* CHA-19: (A) TFH77958 and (B) TFH78995. The phylogenetic tree was obtained by applying a neighbor-joining algorithm to the ClustalW multiple sequence alignment of MCOs. The protein structures of LMCOs were predicted by the ColabFold platform.

MCO is a superfamily of oxidoreductases that catalyze the catalytic oxidation of various substrates, including phenolic compounds and polyphenols such as lignin. LMCO is a specific member of the MCO family that plays an important role in the lignin degradation pathway. Bioinformatics analysis of lignin-degrading bacteria, including RHA1, revealed that their genomes contain putative MCO and DyP genes [32]. CHA-19 cells distinctly expressed two LMCO genes, TFH77958 and TFH78995, upon induction by lignin or lignin-derived vanillic acid (Table 2). This suggests that both genes are involved in the lignin decomposition flux. Based on the classification of MCOs [32], phylogenetic analysis categorized TFH77958 and TFH78995 into groups A and C, respectively (Fig. 5A and 5B). TFH77958 was identified as a group A blue laccase enzyme known to oxidize lignin and its derivatives. Some group C enzymes, likely colorless pseudo-laccases, are known to be involved in copper homeostasis. Compared with studies on the function and diversity of fungal LMCOs, little is known about bacterial LMCOs and their possible implication in lignin decomposition [20].

**Fig. 6:**
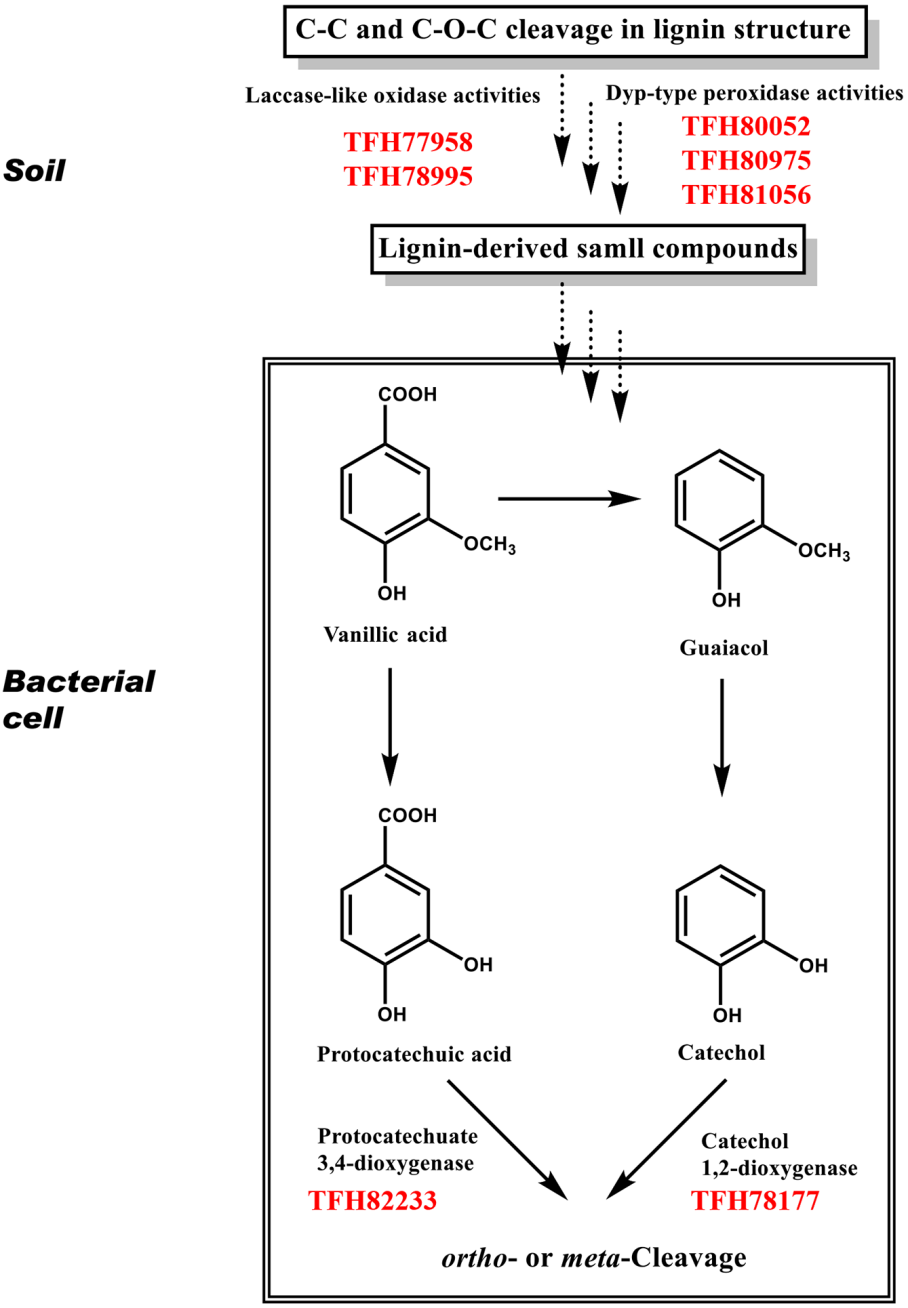
Proposed lignin decomposition pathway (vanillic acid route) in *Pseudomonas kribbensis* CHA-19. Dotted and solid lines represent multistep reactions by different enzymes and one-step reaction by one enzyme, respectively.

In conclusion, the synergistic decomposition of polymeric lignin by a fungal and bacterial consortium appears to occur in natural environments, including cold bipolar tundra soils. Smaller lignin fractions produced through microbial decomposition should be examined in terms of lignin valorization to produce value-added chemicals. Until now, evidence that bacterial enzymes play a more significant role in lignin decomposition than their fungal counterparts has been insufficient and ambiguous. Overall, combined with the fact that CHA-19 can grow on polymeric lignin and lignin-derived small metabolites (phenol, benzoic acid, ferulic acid, and vanillic acid), the spectroscopic data presented here rigorously corroborate the ability of CHA-19 to decompose lignin and lignin model compounds (DDV). CHA-19 seems to prefer LMCOs for initial oxidative lignin decomposition to DyPs, producing smaller metabolites, such as vanillic acid, which is further degraded via a P34O-assisted aromatic ring cleavage pathway (Table 2 and Fig. 6). Studies at the bacterial single-cell level are essential for determining the complexities of lignin decomposition; nevertheless, these approaches have limitations regarding the entire function of bacterial communities in natural environments. However, this study helps to better understand the role of bacteria in lignin decomposition, and thus in terrestrial ecosystems, thereby expanding the biocatalytic toolbox with new bacterial cells and their degradative enzymes for lignin valorization.

## Figures and Tables

**Table 1:** Gene annotation of putative LMCOs & DyPs and neighboring proteins on strain CHA-19 genome.

Gene ID	Length (aa)	Best hit [bacterial or fungal source]^a^	Identity (%)
TFH77956	318	tRNA (mo5U34)-methyltransferase [*Pseudomonas syringae* pv. *tomato*]	253/317 (80%)
TFH77957	165	tRNA-specific adenosine deaminase [*Escherichia coli*]	73/143 (51%)
TFH77958	458	Copper oxidase [*Pseudomonas parafulva*]	356/420 (85%)
TFH77959	1449	Cytotoxic necrotizing factor [*Yersinia pseudotuberculosis*]	85/405 (21%)
TFH77960	525	GMP synthase [*Escherichia coli*]	383/525 (73%)
TFH78993	221	Ribonuclease I [*Escherichia coli*]	47/206 (23%)
TFH78994	160	No significant similarity	
TFH78995	559	Laccase [*Botrytis aclada*]	102/290 (35%)
TFH78996	285	Copper resistance protein B [*Escherichia coli*]	130/215 (60%)
TFH78997	124	Copper resistance protein C [*Pseudomonas syringae*]	82/102 (80%)
TFH78998	287	No significant similarity	
TFH79018	282	Cyclohexadienyl dehydratase [*Pseudomonas aeruginosa*]	120/232 (52%)
TFH80048	554	AMP-dependent synthetase and ligase [*Brucella canis*]	313/544 (58%)
TFH80049	338	AraC family transcriptional regulator [*Pseudomonas aeruginosa*]	34/83 (41%)
TFH80050	282	No significant similarity	
TFH80051	399	Iron uptake system protein EfeO [*Escherichia coli*]	132/324 (41%)
TFH80052	432	Peroxidase EfeB [*Sphingomonas* sp.]	280/380 (74%)
TFH80053	274	Efem M75 Peptidase [*Pseudomonas syringae* pv. *syringae*]	221/255 (87%)
TFH80054	447	Phosphatidylserine synthase [*Haemophilus influenzae*]	215/443 (49%)
TFH80055	188	Putative TetR-family transcriptional regulator [*Streptomyces avermitilis*]	52/169 (31%)
TFH80973	315	No significant similarity	
TFH80974	344	AraC family transcriptional regulator [*Pseudomonas aeruginosa*]	33/84 (39%)
TFH80975	296	Dyp-type peroxidase family protein [*Pseudomonas putida*]	185/289 (64%)
TFH80976	322	1,5-Anhydro-D-fructose reductase [*Sinorhizobium meliloti*]	93/267 (35%)
TFH80977	250	HTH-type transcriptional repressor YvoA [*Bacillus subtilis*]	73/241 (30%)
TFH80978	576	α-Subunit of PAPS reductase [*Methanothermococcus thermolithotrophicus*]	160/539 (30%)
TFH80979	81	β-Subunit of PAPS reductase [*Methanothermococcus thermolithotrophicus*]	20/59 (34%)
TFH80896	262	ROB transcription factor [*Escherichia coli*]	30/99 (30%)
TFH80895	570	Type six secretion system exported effector 8 [*Pseudomonas aeruginosa*]	500/568 (88%)
TFH81056	311	Putative iron-dependent peroxidase [*Streptomyces lividans*]	189/313 (60%)
TFH80894	325	No significant similarity	
TFH80893	366	Polyamine transport protein [*Pseudomonas aeruginosa*]	194/345 (56%)
TFH80892	468	No significant similarity	
TFH80891	161	Flavin reductase-like, FMN-binding protein [*Mycolicibacterium thermoresistibile*]	58/153 (38%)
TFH80890	347	Luciferase-like monooxygenase [*Streptomyces bottropensis*]	98/347 (28%)

^a^The query sequences were searched against Protein Data Bank (PDB) database using NCBI Blastx program.

**Table 2:** Fold changes in mRNA expressions of strain CHA-19 genes involved in the decomposition of lignin and its related compounds.

Induction		TFH80052 (DyP)	TFH80975 (DyP)	TFH81056 (DyP)	TFH77958 (LMCO)	TFH78995 (LMCO)	TFH82233 (P34O)	TFH78177 (C12O)
Time	Substrate							
5-h	Glu	1.0 (± 0.1)^a^	1.0 (± 0.1)	1.0 (± 0.3)	1.0 (± 0.1)	1.0 (± 0.1)	1.0 (± 0.1)	1.0 (± 0.1)
	Van	1.5 (± 0.1)	0.6 (± 0.1)	0.9 (± 0.2)	**2.5 (± 0.1)**	**2.2 (± 0.3)**	**2.5 (± 0.1)**	1.3 (± 0.1)
	Lig	1.9 (± 0.1)	1.0 (± 0.1)	1.4 (± 0.7)	**3.4 (± 0.4)**	**3.7 (± 0.8)**	**3.1 (± 0.6)**	1.8 (± 0.1)
24-h	Glu	1.0 (± 0.2)	1.0 (± 0.0)	1.0 (± 0.0)	1.0 (± 0.2)	1.0 (± 0.0)	1.0 (± 0.1)	1.0 (± 0.1)
	Van	2.2 (± 1.0)	1.8 (± 0.1)	0.9 (± 0.1)	1.8 (± 0.1)	**2.6 (± 0.3)**	**2.1 (± 0.3)**	1.7 (± 0.1)
	Lig	**6.4 (± 1.8)**	**5.4 (± 0.1)**	**2.0 (± 0.5)**	**5.6 (± 0.7)**	**7.9 (± 0.9)**	**6.5 (± 0.4)**	**3.7 (± 0.3)**

^a^The fold change values from qPCR results are mean (± standard deviation) of three technical replicate. Higher than 2.0-fold changes are highlighted in bold. Abbreviations: Glu, glucose; Van, vanillic acid; Lig, lignin.
